# A Naturalistic, Non-interventional Investigation of the Clinical and Sociodemographic Characteristics and Prescription Patterns in Patients With Psychotic Disorders at a Tertiary Care Facility in South Asia

**DOI:** 10.7759/cureus.53541

**Published:** 2024-02-04

**Authors:** Zarrin Ansari, Abhilasha Rashmi, Sudhir Pawar

**Affiliations:** 1 Pharmacology and Therapeutics, Lokmanya Tilak Municipal Medical College and General Hospital, Mumbai, IND

**Keywords:** prevalence, psychotic disorder, polypharmacy, prescription pattern, antipsychotics

## Abstract

Objectives: This study aimed to evaluate the trends in the sociodemographic, clinical, and prescription characteristics of patients with psychotic illnesses seen in the outpatient psychiatry department of a tertiary care facility.

Methods: Between March 2021 and April 2022, a cross-sectional, prospective, observational, naturalistic, non-interventional study was conducted. A total of two hundred prescriptions were analyzed. To assess the rationality of prescriptions, World Health Organization (WHO) indicators were also computed.

Results: With a range of 18 to 75 years, the cohort's mean age was 40.26 years, and its average disease duration was 10.75 years. Sixty-seven patients (68.5%) were diagnosed with schizophrenia. Of the 200 prescriptions that were analyzed, 13 antipsychotic prescriptions were written 343 times. Olanzapine was prescribed as an antipsychotic the most frequently (132, 66%), followed by clozapine (75, 37.5%). Haloperidol (41, 20.5%), trifluoperazine (3, 1.5%), loxapine (1, 0.5%), and flupenthixol depot (1, 0.5%) were the most commonly prescribed typical antipsychotics. 91% (181/200) of patients received prescriptions for other drugs in addition to antipsychotics. Trihexyphenidyl (45%), escitalopram (30%), clonazepam (26.5%), sodium valproate (10%), propranolol (10.5%), and modafinil (9.5%) were the most frequently prescribed concurrent medicines. Forty-eight percent (95/200) of prescriptions demonstrated polypharmacy. Among patients, the frequency of antipsychotic prescriptions was 1 in 44% (88/200), 2 in 36.50% (73/200), 3 in 17% (34/200), 4 in 0.5% (1/200), and 5 again in 0.5% (1/200).

Conclusions: On average, the cohort of the current study was young. The commonest diagnosis was mainly schizophrenia. Atypical antipsychotics accounted for the majority of antipsychotic prescriptions in the current study. In this study, a high prevalence of polypharmacy was noted.

## Introduction

Psychosis is a debilitating cluster of symptoms characterized by delusions, hallucinations, disorganized thinking and speech, and negative and cognitive symptoms as defined by the Diagnostic and Statistical Manual of Mental Disorders, Fifth Edition (DSM-5) [1,2]. Patients afflicted with psychosis lose touch with reality. Psychosis can be a presenting symptom of many psychiatric, neurodevelopmental, neurologic, and medical conditions. The lifetime prevalence of psychotic disorders was reported to be 3.06% in the general population [3]. Psychosis is a hallmark symptom of schizophrenia spectrum disorders which include schizophrenia, schizoaffective disorders, delusional disorders, brief psychotic disorders, and psychotic disorders not otherwise specified. Psychosis can also be associated with; affective disorders (bipolar I disorder and major depressive disorder, that is, MDD), substance abuse disorder, and general medical conditions like dementia [1,3].

Psychotropic medications share a cardinal role in the management of the aforementioned disorders. Despite the availability of national and international guidelines guiding the prescriptions in psychotic disorders, there are several evidence of the irrationality of prescriptions. There has been documented evidence of polypharmacy in the treatment of psychiatric patients. The use of multiple medications may increase the risk of adverse effects, drug interactions, non-compliance, and medication errors. In a considerable proportion of patients, polypharmacy is a requisite and rational. For example, a prescription of anticholinergics is considered rational when prescribed to counter the adverse events of antipsychotics [4,5].

The principal aim of prescription pattern studies is to facilitate the rational use of drugs in populations. Therefore, it is crucial to conduct regular research on drug use patterns in different hospital settings or patient demographics in order to evaluate the hospital's current drug policies critically and, if necessary, offer recommendations based on various principles for improving the pattern of drug use going forward. This study focuses on analyzing the prescribing pattern of drugs in patients with psychotic disorders.

This article was previously presented as a meeting abstract at the 74th Annual National Conference of Indian Psychiatric Society (ANCIPS 2023) on February 2, 2023. 

## Materials and methods

In this study, the authors have carefully observed and recorded the prescription pattern cross-sectionally, in its natural setting while interfering as little as possible with the subjects and their disorders. This was designed as a prospective, non-interventional, naturalistic, cross-sectional, observational study at a tertiary care center in urban India. The ethics committee approval (IEC/255/21) was obtained on March 2, 2021, from the institutional ethics committee of the Lokmanya Tilak Municipal Medical College and General Hospital, Mumbai. Data collection was conducted between March 2021 and April 2022 by visiting the psychiatry outpatient department every day. All adult patients (>/= 18 years) of either sex, diagnosed with psychotic disorders according to DSM-5, and stable on medications were included in the analysis. Diagnoses of the psychotic disorders were done by qualified psychiatrists practicing in the outpatient department. All patients with acute episodes of psychosis were excluded. Patients were explained about the non-interventional nature of the study and were assured about preserving their identities before enrolment. Written informed consent was obtained from all of the participants of the study. A total of 200 patient prescriptions that met the inclusion criteria were analyzed. A convenience sampling method was adopted to recruit participants. 

The data collected included demographic characteristics, viz., age, gender, employment status, marital status, educational status, and disease data including the duration of illness. Prescription data included prescription name, dose, frequency of administration, dosage form, route of administration of antipsychotics, and concomitant psychotropic and non-psychotropic medications. The drugs were grouped as per the 21st WHO Essential Medicines List (EML) updated in June 2019 [6]. The institute’s hospital pharmacy was consulted to check whether the prescriptions were included in the hospital formulary list.

After analysis of the prescription pattern, the following WHO indicators were calculated to understand the rationality of the prescriptions: average number of drugs prescribed per encounter, percentage of antipsychotics prescribed, percentage of other classes of drugs, types of dosage forms prescribed, percentage of drugs prescribed by generic name, percentage of drugs prescribed from WHO EML and percentage of drugs prescribed from hospital formulary.

Statistical analyses were conducted using Microsoft Excel for Mac (Version 15.13.3-150815) and the Statistical Package for Social Sciences (SPSS), version 25.0 (IBM Corp., Armonk, NY). Categorical variables were described in terms of proportions and continuous variables in terms of mean, median, standard deviation, and range. The study data was mainly analyzed by descriptive statistics. A chi-square test of association was performed along with logistic regression (entry method).

## Results

Patient’s sociodemographic and clinical characteristics

A total of 200 prescriptions of patients with psychotic disorders were analyzed between March 2021 and April 2022. The cohort's age ranged from 18 to 75 years, with a mean of 40.26 [±12.948] years. The range of the illness duration was 1-40 years, with a mean of 10.75 [±8.612] years. There were 116 patients aged between 18-40 years and 84 patients between 41-75 years of age.

More males (122, 61%) were found to have psychotic disorders than females (78, 39%). Seventy-one (35.5%) of male patients were financially independent compared to only 9 (4.5%) of female patients. Financially independent patients included those who were employed and retired with pension and the dependent included unemployed and housewives.

Just 21% (42/200) of the patients had completed college or higher education; of these, 31 (15.5%) were men and only 11 (5.5%) were women. Out of 200, 79% had completed at least one year of school. Of the patients, 58% were married, while 42% were separated, divorced, single, or widowed. One hundred and seventeen out of 200 (58.5%) patients had a history of hospitalization due to their psychiatric ailment.

Table 1 elaborates on the various psychotic disorders among the 200 patients analyzed. One hundred and thirty-seven (68.5%) patients were diagnosed with schizophrenia without any comorbid psychiatric conditions. An additional 19% (38/200) were diagnosed with schizophrenia plus neuropsychiatric comorbidities. The most common neuropsychiatric comorbidities were substance use disorders (19/200, 9.5%) and epilepsy (12/200, 6%). Twelve (6%) patients had non-neuropsychiatric comorbidities, viz., ischemic heart diseases, hypertension, diabetes mellitus, hypothyroidism, asthma, polyradiculopathy, and adenomyosis of the uterus. These were being managed by their respective specialties.

**Table 1:** Frequency of various psychotic disorders OCD: Obsessive-compulsive disorder, MDD: Major depressive disorder

Diagnosis	Frequency (n)	Percentage (%)
Schizophrenia	137	68.5
Schizoaffective disorder	4	2
Schizoaffective disorder + epilepsy disorder	1	0.5
Schizophrenia + alcohol and nicotine use disorder	1	0.5
Schizophrenia + alcohol use disorder	9	4.5
Schizophrenia + cannabis use disorder	1	0.5
Schizophrenia + epilepsy disorder	10	5
Schizophrenia + hypochondriasis	1	0.5
Schizophrenia + nicotine use disorder	3	1.5
Schizophrenia + OCD	3	1.5
Schizophrenia + OCD + epilepsy disorder	1	0.5
Schizophrenia + OCD + nicotine use disorder	1	0.5
Schizophrenia + post-psychotic depression	6	3
Schizophrenia + migraine (headache)	2	1
Delusional disorder	4	2
Psychotic features + bipolar disorder	1	0.5
Psychotic features + cannabis use disorder	1	0.5
Psychotic features + intellectual disability	3	1.5
Psychotic features + MDD	7	3.5
Psychotic features + MDD + nicotine use disorder	1	0.5
Psychotic features + MDD + panic disorder	1	0.5
Psychotic features + MDD + polysubstance use disorder	1	0.5
Psychotic features + polysubstance use disorder	1	0.5
Total (N)	200	100

Antipsychotic prescriptions

Thirteen antipsychotics were prescribed 343 times out of the 200 prescriptions that were examined. The most often prescribed antipsychotic was olanzapine (132/200, 66%), which was followed by clozapine (75/200, 37.5%). Only once a depot of flupenthixol was prescribed and the rest of the prescriptions included oral dosage forms. Haloperidol (41/200, 20.5%), trifluoperazine (3/200, 1.5%), loxapine (1/200, 0.5%), and flupenthixol depot (1/200, 0.5%) were the most common typical antipsychotics administered. Typical antipsychotics formed a total of 23% of antipsychotic prescriptions. The ranking of antipsychotics use was as follows: olanzapine (66%), clozapine (37.5%), haloperidol (20.5%), risperidone (13%), blonanserin (11.5%), quetiapine (9%), aripiprazole (5.5%), amisulpride (4%), trifluoperazine (1.5%), tiapride (1.5%), loxapine (0.5%), flupenthixol depot (0.5%) and ziprasidone (0.5%) (Table 2).

**Table 2:** Frequency and percentage of antipsychotic drugs Frequency is denoted by the letter "n" in this table

Frequency and percentage of antipsychotics			Dosage	
	Percentage (%)	Frequency (n)	Range (mg/day)	Average (mg/day)
Olanzapine	66	132	2.5 - 20 mg/day	13.3 mg/day
Clozapine	37.5	75	10 - 400 mg/day	111.8 mg/day
Haloperidol	20.5	41	2.5 - 20 mg/day	11 mg/day
Trifluoperazine	1.5	3	5 - 10 mg/day	8.33 mg/day
Risperidone	13	26	1 - 8 mg/day	3.96 mg/day
Quetiapine	9	18	25 -100 mg/day	66.67 mg/day
Loxapine	0.5	1	25 mg/day	25 mg/day
Tiapride	1.5	3	50 - 300 mg/day	141.67 mg/day
Blonanserin	11.5	23	8 - 24 mg/day	13.60 mg/day
Aripiprazole	5.5	11	5 - 20 mg/day	13.33 mg/day
Amisulpride	4	8	50 - 600 mg/day	206.25 mg/day
Flupenthixol depot	0.5	1	20 mg/week	20 mg/week
Ziprasidone	0.5	1	40 mg/day	40 mg/day

When the chi-square test of association was performed between clozapine and other demographic variables, clozapine was found to be significantly associated with variables, viz., gender (0.001), history of hospitalization (0.007), and education of subjects (0.009). Similarly, olanzapine was found to be significantly associated with the status of education (0.014), risperidone was associated with the history of hospitalization (0.026), and tiapride with marital status (0.011). The tests of association with other antipsychotics and demographic variables did not show statistically significant results.

Concomitant medications

Apart from antipsychotics, 91% (181/200) of patients received concurrent prescriptions for other drugs. Fifty-eight non-antipsychotic concomitant drugs were prescribed 589 times in the 200 analyzed prescriptions. These prescriptions included both psychotropic as well as non-psychotropic medications. The common groups of concomitant psychotropic medications included antidepressants, sedatives/hypnotics, anti-epileptics, mood stabilizers, drugs for migraine/headache, sleep cycle regulators, drugs for dementia, drugs for substance abuse, drugs for extrapyramidal syndrome (EPS) and restless leg syndrome (RLS). Trihexyphenidyl (45%), escitalopram (30%), clonazepam (26.5%), sodium valproate (10%), propranolol (10.5%), and modafinil (9.5%) were the most frequently prescribed concurrent medicines. The most often given non-psychotropic drugs were calcium, iron supplements, and multivitamin B complex (52%) (Table 3).

**Table 3:** Frequency and percentage prescription of drugs other than antipsychotics MVBC: Multivitamin B-complex, FSFA: Ferrous sulfate/folic acid, RLS: Restless leg syndrome, EPS: Extrapyramidal syndromes, NA: Not applicable

	Other drugs	Frequency (n)	Percentage (%)	Dose range (mg/day)	Average dose (mg/day)
Antidepressants	Mirtazapine	13	6.5	15 - 45 mg/day	25.77 mg/day
	Fluoxetine	9	4.5	40 - 90 mg/day	54.44 mg/day
	Clomipramine	6	3	25 - 150 mg/day	56.25 mg/day
	Paroxetine	6	3	12.5 - 50 mg/day	37.50 mg/day
	Desvenlafaxine	3	1.5	100 - 200 mg/day	166.67 mg/day
	Escitalopram	60	30	5 - 30 mg/day	15.33 mg/day
	Imipramine	7	3.5	25 - 150 mg/day	57.14 mg/day
	Amitriptyline	9	4.5	25 - 100 mg/day	48.61 mg/day
	Venlafaxine	3	1.5	75 - 225 mg/day	125 mg/day
	Fluvoxamine	1	0.5	50 mg/day	50 mg/day
	Nortriptyline	1	0.5	75 mg/day	75 mg/day
	Vilazodone	1	0.5	40 mg/day	40 mg/day
Sedative/ Hypnotics	Clonazepam	53	26.5	0.25 -2 mg/day	0.67 mg/day
	Oxazepam	3	1.5	20 - 45 mg/day	31.67 mg/day
	Lorazepam	8	4	0.5 - 4 mg/day	2.19 mg/day
	Clobazam	1	0.5	20 mg/day	20 mg/day
	Etizolam	1	0.5	0.5 mg/day	0.5 mg/day
	Alprazolam	2	1	1 - 1.5 mg/day	1.25 mg/day
Anti-epileptics	Levetiracetam	1	0.5	1500 mg/day	1500 mg/day
	Phenobarbital	3	1.5	80 - 180 mg/day	126.67 mg/day
	Phenytoin	7	3.5	100 - 300 mg/day	228.57 mg/day
	Carbamazapine	3	1.5	400 - 1200 mg/day	666.67 mg/day
	Oxcarbazapine	1	0.5	600 mg/day	600 mg/day
	Topiramate	2	1	50 mg/day	50 mg/day
Mood stabilizers	Lithium	6	3	400 - 1200 mg/day	733.33 mg/day
	Divalproate	8	4	500 - 1000 mg/day	656.25 mg/day
	Lamotrigine	4	2	50 - 200 mg/day	137.50 mg/day
	Sodium valproate	20	10	200 - 1400 mg/day	600 mg/day
Drugs for migraine/headache	Flunarizine	3	1.5	5 - 20 mg/day	11.67 mg/day
	Naproxane	1	0.5	250 mg/day	250 mg/day
	Domperidone	1	0.5	10 mg/day	10 mg/day
	Rizatriptan	1	0.5	10 mg/day	10 mg/day
Sleep cycle regulators	Modafinil	19	9.5	50 - 200 mg/day	118.42 mg/day
	Armodafinil	3	1.5	150 mg/day	150 mg/day
	Melatonin	1	0.5	3 mg/day	3 mg/day
Drugs for dementia	Donepezil	5	2.5	5 - 10 mg/day	7 mg/day
	Memantine	1	0.5	5 mg/day	5 mg/day
Drugs for substance abuse drugs	Acamprosate	7	3.5	999 - 1998 mg/day	1379.57 mg/day
	Naltrexone	1	0.5	50 mg/day	50 mg/day
	Disulfiram	1	0.5	500 mg/day	500 mg/day
	Buspirone	1	0.5	15 mg/day	15 mg/day
	Baclofen	6	3	30 - 60 mg/day	35 mg/day
RLS	Pramipexole	3	1.5	0.125 - 0.5 mg/day	0.29 mg/day
Drugs for EPS and related symptoms	Tetrabenazine	2	1	25 - 50 mg/day	37.50 mg/day
	Trihexyphenidyl	90	45	1 - 6 mg/day	3.43 mg/day
	Propranolol	21	10.5	20 - 60 mg/day	24.76 mg/day
Others	MVBC/FSFA	73	36.5	NA	NA
	Calcium	31	15.5	500 - 1000 mg/day	532.26 mg/day
	Metformin	18	9	500 - 1500 mg/day	888.89 mg/day
	Bisacodyl	18	9	5 - 10 mg/day	7.78 mg/day
	Amlodipine	6	3	2.5 - 10 mg/day	6.25 mg/day
	Ondansetron	1	0.5	4 mg/day	4 mg/day
	Ranitidine	2	1	300 mg/day	300 mg/day
	Vitamin E	1	0.5	800 mg/day	800 mg/day
	Pantoprazole	21	10.5	40 - 80 mg/day	41.90 mg/day
	Glycopyrrolate	2	1	1 -2 mg/day	1.5 mg/day
	Cerebroprotein hydrolysate	1	0.5	90 mg/day	90 mg/day
	Paracetamol	6	3	500 mg/day	500 mg/day

Chi-square tests and odds ratio (OR) were performed to check the association of the three commonest concomitant drugs with the antipsychotic prescription. Results of association of antipsychotics which tested significantly positive with the three concomitant drugs are tabulated along with their respective ORs. The odds of prescribing trihexyphenidyl with haloperidol were found to be 98% (Table 4).

**Table 4:** Chi-square table of association between antipsychotics and other psychotropic drugs The p value at which results were considered significant were *p<0.05 and **p<0.001

List of psychotic drugs		Trihexyphenidyl		Total	p value	Odds ratio (OR)
		Yes	No			
Olanzapine	Yes	50	82	132	0.042*	0.542
	No	36	32	68		
Haloperidol	Yes	40	1	41	0.0001**	98.261
	No	46	113	159		
Risperidone	Yes	23	3	26	0.0001**	13.508
	No	63	111	174		
Blonanserin	Yes	17	6	23	0.001**	4.435
	No	69	108	177		
		Escitalopram		Total		
		Yes	No			
Clozapine	Yes	13	62	75	0.003**	0.360
	No	46	79	125		
Haloperidol	Yes	5	36	41	0.006**	0.270
	No	54	105	159		
		Clonazepam		Total		
		Yes	No			
Clozapine	Yes	26	49	75	0.043*	1.926
	No	27	98	125		

Fixed dose combinations (FDCs)

The prescriptions included five FDCs, viz., risperidone plus trihexyphenidyl (1%), trifluoperazine plus trihexyphenidyl (1.5%), clonazepam plus propranolol (5%), naproxen plus domperidone (0.5%) and donepezil plus memantine (0.5%).

Polypharmacy

In the current study, just 6% of prescriptions contained a single medication (Table 5). For the present study, we adopted the WHO definition of polypharmacy as the use of five or more medications to treat one patient. Consequently, 47.5% (95/200) of prescriptions demonstrated polypharmacy. Among patients, the frequency of antipsychotic prescriptions was 1 in 44% (88/200), 2 in 36.50% (73/200), 3 in 17% (34/200), 4 in 0.5% (1/200), and 5 again in 0.5% (1/200). Antipsychotics were not prescribed in any of the 3/200 (1.5%) prescriptions. The diagnoses mentioned in these three prescriptions were MDD with psychotic features in one patient and schizophrenia with post-psychotic depression in two patients. These three patients were mainly treated with amitriptyline, escitalopram, vilazodone, divalproate, and modafinil. The ratio of atypical antipsychotics to typical antipsychotic prescriptions is elaborated in Table 6. 

**Table 5:** Frequency of total drugs per prescription

Total number of drugs per prescription	Frequency (n)	Percentage (%)
1	12	6
2	26	13
3	26	13
4	41	20.5
5	24	12
6	29	14.5
7	21	10.5
8	9	4.5
9	7	3.5
10	4	2
11	1	0.5

**Table 6:** Frequencies of typical and atypical antipsychotic prescriptions

Prescription pattern of antipsychotic drugs	Number of prescriptions
1 Atypical	4
1 Atypical : 1 Typical	20
2 Atypical : 1 Typical	19
2 Atypical	51
3 Atypical	14
4 Atypical	1
4 Atypical: 1 Typical	1

WHO indicators

The results of WHO indicators are tabulated (Table 7). When calculating the WHO indicator, the FDCs were considered as separate drugs for all parameters except the percentage of drugs prescribed by brand and generic names.

**Table 7:** WHO indicators WHO EML: World Health Organization Essential Medicines List

Drug use indicators	Results
Average number of drugs prescribed per encounter	4.66
Percentage of antipsychotic drugs prescribed	343/932 X 100 = 36.8%
Percentage of other classes of drugs prescribed	589/932 X 100 = 63.2%
Percentage of oral dosage forms prescribed	931/932 X 100 = 99.89%
Percentage of depot dosage forms prescribed	1/932 X 100 = 0.11%
Percentage of drugs prescribed by generic name	629/915 X 100 = 68.74%
Percentage of drugs prescribed by brand name	285/915 X 100 = 31.14%
Percentage of drugs prescribed from available WHO EML	19/71 X 100 = 26.76%
Percentage of drugs prescribed from available hospital drugs formulary	24/71 X 100 = 33.8%

## Discussion

In the present study involving 200 patients, 68.5% were diagnosed with schizophrenia without any neuropsychiatric comorbid conditions. An additional 19% (38/200) were diagnosed with schizophrenia plus neuropsychiatric comorbidities. Thus, the total prevalence of schizophrenia in the present study was 87.5% among all psychotic disorders. This was in contrast to the study conducted by Perälä et al. which reported a prevalence of schizophrenia alone of 27% (67/249) [3]. The commonest comorbidity with schizophrenia in the present study was substance use disorder 7.5% (15/200). The prevalence of schizoaffective disorder was 2.5% (5/200), delusional disorder was 2% (4/200), MDD with psychotic features was 5% (10/200), and bipolar disorder with psychotic features was 0.5% (1/200) in the present study. Perälä et al. reported the prevalence of schizoaffective disorder as 10% (24/249), delusional disorder as 6% (15/249), MDD with psychotic features as 12% (29/249) and bipolar disorder with psychotic features as 8% (20/249) [3]. The discrepancy in the prevalence between the two studies could be because of the difference in the diagnostic manuals used by the two different setups. Perälä et al. used DSM-IV as a diagnostic reference as their study was conducted in the year 2007 and the center for the present study used DSM-5 as a reference diagnostic manual [3].

All antipsychotic prescriptions represented 98.5% of all outpatient prescriptions, atypical antipsychotics represented 87%, and the typical represented 13% of all antipsychotic prescriptions in the current study. This is in alignment with the current treatment guidelines which in general recommend the use of atypical antipsychotics as first-line medications in the treatment of schizophrenia and other psychotic disorders [7-10]. Atypical antipsychotics have demonstrated a marked improved safety profile in terms of lesser extrapyramidal and endocrine adverse events as compared to typical antipsychotics, thereby improving quality of life and improving patient’s willingness to adhere to maintenance treatment. Not just a markedly improved safety profile, atypical antipsychotics also demonstrate more effectiveness across a broader range of symptoms of psychosis than typical antipsychotics [11]. In the United States (US), Aparasu et al. demonstrated that the proportion of visits for atypical antipsychotics, as a percentage of visits for all antipsychotic drugs, rose sharply from about 48% in 1998 to 84% in 2002 [12]. Correspondingly, the percentage of visits involving first-generation antipsychotic drugs declined [12]. In a Norwegian study conducted by Johnsen et al., it was reported that 82/149 patients used a single antipsychotic and 65% of these received atypical antipsychotics [13]. Atik et al. from Turkey demonstrated that atypical antipsychotics represented 75.1% and typical antipsychotics represented 24.9% of the antipsychotic prescriptions in a retrospective chart review [14]. Similarly, Centorrino et al. from France reported that 88% percentage of antipsychotics prescribed were atypical [15]. From India, Ramadas et al. demonstrated that 59% of patients with schizophrenia received atypical antipsychotics and only 10% of patients received typical antipsychotics [16]. In contrast to the above findings, Bret et al. reported that atypical antipsychotics accounted for 43.2% of prescriptions and typical antipsychotics accounted for 56.8% of them [17]. 

The choice of antipsychotics also has an impact on the overall outcome in patients with psychotic disorders. In the present study, olanzapine was the highest prescribed antipsychotic (66%) followed by clozapine (37.5%), haloperidol (20.5%), risperidone (13%), and blonanserin (11.5%). It is worth noting that the relatively newer antipsychotic, blonanserin, approved recently by the Japanese regulatory body Pharmaceuticals and Medical Devices Agency (PhMDA), formed 11.5% of the total antipsychotic prescriptions. Studies from the US by Hermann et al., United Kingdom by Ashcroft et al., France by Boulin et al., Centorrino et al., and Bret et al., Italy by Mauri et al., India by Grover et al., and Korea by Kim et al. reported more frequent use of olanzapine and risperidone than other antipsychotics [17-24]. 

Though overall, the atypical antipsychotics scored above the typical antipsychotics in terms of the frequency of prescription, haloperidol still occupied 20.5% of total antipsychotic prescriptions in the present study. Rosenheck et al. evaluated the cost and effectiveness of olanzapine versus haloperidol in a randomized controlled trial (RCT) [25]. It was demonstrated that there were no significant differences between groups in study retention; positive, negative, or total symptoms of schizophrenia; quality of life; or extrapyramidal symptoms. Olanzapine did not demonstrate advantages compared with haloperidol in compliance, symptoms, extrapyramidal symptoms, or overall quality of life, and its benefits in reducing akathisia and improving cognition seemed to be balanced with the problems of weight gain and higher cost [25]. Clozapine occupied 37.5% of antipsychotic prescriptions mainly for the management of resistant schizophrenia with or without comorbidity (35.5%). Four cases (2%) of clozapine prescriptions were administered for the diagnoses of schizoaffective disorder, intellectual disability with psychotic features, and delusional disorder. This is in alignment with the recommendation made by the American Psychiatric Association guidelines (APA) to use clozapine for treatment-resistant schizophrenia and in patients with the risk of aggressive behavior [7]. 

In almost 60% of cases, at least one antidepressant was prescribed, and escitalopram formed 50% of this prescription with 60/200 frequency in the present study. When depression was investigated longitudinally in schizophrenia, 80% of patients experienced a clinically significant depressive episode at one or more time points during the early phase of the disease [26]. The considerable usage of antidepressants in the present study reflects a significant presence of depression or depressive episodes in patients suffering from psychotic disorders. In the present study, 10% of patients were prescribed sodium valproate as a mood stabilizer mainly to treat the affective symptoms developed in patients with psychotic disorders. Clonazepam formed 26.5% of prescriptions, propranolol formed 10% of the prescriptions and in 7.5% of prescriptions, clonazepam and propranolol were prescribed together. These were prescribed for antipsychotic-induced akathisia and associated anxiety and tremors. Anticholinergics, viz., trihexyphenidyl was prescribed in 45% of patients to balance the extrapyramidal adverse effects of the antipsychotics. Two patients were prescribed tetrabenazine, a reversible inhibitor of the vesicular monoamine transported 2 (VMAT-2), for tardive dyskinesia. These psychotropic co-prescriptions were in alignment with the current treatment guidelines [8,27].

Amongst the non-psychotropic co-prescriptions, metformin was prescribed in 9% of non-diabetic patients in the present study. This off-label prescription was mainly to counter the weight gain associated with antipsychotics. Sialorrhea is a common and troublesome adverse effect seen with clozapine which leads to poor compliance [28]. In the present study glycopyrrolate, an anti-cholinergic, was prescribed to counter this adverse event. Despite modern electroconvulsive therapy (ECT) techniques, cognitive side-effects remain an important issue, although their nature and degree remain to be clarified fully [29]. Cerebroprotein hydrolysate has been attempted to improve post-ECT cognitive impairments with modest effects in the present study.

Our results demonstrated that there was widespread polypharmacy in patients with psychotic disorders. Average number of drugs prescribed per prescription was 4.66. Only 6% of prescriptions showed a single drug prescribed and the rest 94% had medications ranging from 2-11. A study conducted by Grohmann et al. observed that polypharmacy was reported in about three-quarters of patients on psychotropic drugs, with a trend towards increasing use over time [30]. 

As per the calculated WHO indicators, the use of depot formulations of antipsychotics was markedly low in the current study. The most important reason for this scanty usage was the cost of the depot formulations. Depots were not part of the hospital formulary and thus the cost had to be borne by the patients. This largely discouraged the usage of depot formulation at this center. 

Limitations of the study

This study was conducted at a single center and thus could represent biased results. Since the study was conducted at a tertiary care hospital, it is not representative of a pan-Indian population which involves a large section of rural set-up. The cross-sectional nature of the study simply eliminates observation of longitudinal time-wise change in the pattern of prescription. Finally, the present study included only outpatient stable patients and thus did not represent a complete sample of acutely ill with high-severity inpatients. The prescription pattern, diagnosis, and severity in the inpatient sample markedly differ from the outpatient sample.

## Conclusions

The study revealed that the majority of antipsychotic prescriptions were for atypical antipsychotics. This new class of antipsychotics is being used more frequently, which emphasizes the need for a more thorough assessment of their safety profile. Another finding of this study was the high occurrence of polypharmacy. Enormous numbers of co-prescriptions to antipsychotics were reported in this study. These included prescriptions to treat the coexisting disorders and to balance the adverse events of antipsychotics. These co-prescriptions, especially off-label ones, need careful evaluation and further research.
